# Improving Pain Assessment Using Vital Signs and Pain Medication for Patients With Sickle Cell Disease: Retrospective Study

**DOI:** 10.2196/36998

**Published:** 2022-06-23

**Authors:** Swati Padhee, Gary K Nave Jr, Tanvi Banerjee, Daniel M Abrams, Nirmish Shah

**Affiliations:** 1 Department of Computer Science and Engineering, Wright State University Dayton, OH United States; 2 Department of Engineering Sciences and Applied Mathematics, Northwestern University Chicago, IL United States; 3 Division of Hematology, Duke University School of Medicine Durham, NC United States

**Keywords:** pain management, pain medication, vital signs, sickle cell disease, machine learning

## Abstract

**Background:**

Sickle cell disease (SCD) is the most common inherited blood disorder affecting millions of people worldwide. Most patients with SCD experience repeated, unpredictable episodes of severe pain. These pain episodes are the leading cause of emergency department visits among patients with SCD and may last for several weeks. Arguably, the most challenging aspect of treating pain episodes in SCD is assessing and interpreting a patient’s pain intensity level.

**Objective:**

This study aims to learn deep feature representations of subjective pain trajectories using objective physiological signals collected from electronic health records.

**Methods:**

This study used electronic health record data collected from 496 Duke University Medical Center participants over 5 consecutive years. Each record contained measures for 6 vital signs and the patient’s self-reported pain score, with an ordinal range from 0 (no pain) to 10 (severe and unbearable pain). We also extracted 3 features related to medication: *medication type*, *medication status (given or applied*, or *missed or removed or due)*, and *total medication dosage (mg/mL)*. We used variational autoencoders for representation learning and designed machine learning classification algorithms to build pain prediction models. We evaluated our results using an accuracy and confusion matrix and visualized the qualitative data representations.

**Results:**

We designed a classification model using raw data and deep representational learning to predict subjective pain scores with average accuracies of 82.8%, 70.6%, 49.3%, and 47.4% for 2-point, 4-point, 6-point, and 11-point pain ratings, respectively. We observed that random forest classification models trained on deep represented features outperformed models trained on unrepresented data for all pain rating scales. We observed that at varying Likert scales, our models performed better when provided with medication data along with vital signs data. We visualized the data representations to understand the underlying latent representations, indicating neighboring representations for similar pain scores with a higher resolution of pain ratings.

**Conclusions:**

Our results demonstrate that medication information (the type of medication, total medication dosage, and whether the medication was given or missed) can significantly improve subjective pain prediction modeling compared with modeling with only vital signs. This study shows promise in data-driven estimated pain scores that will help clinicians with additional information about the patient’s condition, in addition to the patient’s self-reported pain scores.

## Introduction

### Background

Sickle cell disease (SCD) is a family of genetic blood disorders that affects >20 million people worldwide [1], the most prevalent complication of which is pain. Pain crises in SCD are strongly linked to increased medical costs, morbidity, and mortality [2]. During childhood, SCD often presents as unpredictable, severe, acute pain episodes characterized by pain periods ranging from hours to weeks, which usually occur a few times a year. The most challenging aspect of treating pain episodes in SCD is the assessment and interpretation of the patient’s pain intensity level [3,4]. However, in current clinical practice, patient self-reporting is the gold standard approach to determining the absence, presence, and severity of pain [4,5].

Furthermore, because of the subjective nature of pain, it is challenging for clinicians to precisely ascertain the severity of the patient’s pain. This assessment is particularly difficult in patients with chronic pain. Furthermore, effective treatment strategies for patients with SCD, such as intravenous opioid therapy, are palliative. Ultimately, as pain is inherently subjective, medical providers and patients have difficulty in determining the ideal treatment and management strategies for pain. As a result, there has been an increasing focus on developing and implementing pain prediction models from objective measures over the past several years [3,4,6-8]. However, in addition to the slow development of these models, the difficulty also lies in understanding the severity of a patient’s pain level and their response to pain management strategies.

Currently, the standard treatment protocol for painful episodes associated with SCD includes rest, aggressive hydration, treatment of any underlying infections or other complications, and a focus on analgesics such as opioids [9,10]. However, there is wide variability in the management of painful episodes in hospitals. Variations in practice reflect different views about the suitability of opioids, such as concerns of dependence on opioids. In addition, each patient has historical differences in responses to opioids; the methods of administration of opioids, such as continuous infusion and patient-controlled analgesia, lead to varied responses in patients. Pharmacological management strategies for acute pain associated with SCD include opioids, nonopioids, and adjuvant analgesics or coanalgesics. Medication strategies for chronic pain are diverse and lead to substantial variability. Hence, it is essential to consider the inclusion of pain medication when modeling pain prediction.

The current literature shows an increasing focus on machine learning (ML) techniques to understand the various complexities associated with patient health in SCD [11-14]. Lazakidou et al [15] developed a personal electronic health record (EHR) to evaluate the deployment of an advanced web-based application platform that assessed health care professionals and patients to provide a more efficient and effective solution than that of the daily clinical routine. In their study, web-based solutions enabled patients to update and access their medical information. The system was examined with 3 varied patient groups comprising 150 patients with Parkinson disease, diabetes, and congenital heart disease engaged in 3 European clinics. The outcomes indicated that personal EHRs could provide better services in terms of user-friendliness, data management, comprehensiveness, and valuable content. Du et al [16] developed a microfluidic device that could examine the behavior of blood from patients with SCD. This device could also measure how long blood cells took to become stiff and get stuck in the blood vessels. A total of 25 patients with SCD were involved in their study. By using this device to evaluate blood samples, the researchers were able to decide how deoxygenation affected the sickling rates of red blood cells (RBCs), capillary’ stick rates, and how quickly the RBCs reshaped, especially when oxygen levels were restored. Knowlton et al [17] presented a sensitive, label-free, and specific testing platform to diagnose SCD using blood samples based on the density of sickle RBCs under deoxygenated conditions. Using this platform, they could differentiate between the levitation patterns of sickle and control RBCs in association with their degree of confinement.

In the face of the continued opioid crisis, the search for more objective measures of pain continues to evolve rapidly in medicine, and studies examining a variety of objective measures to predict pain have been published in recent years [7,18]. Prior studies have reported preliminary evidence that fluctuations in vital signs may be used to assess pain in patients in the intensive care unit [19] as acute pain leads to changes in vital signs [20]. These physiological measures include blood pressure, respiratory rate, oxygen saturation, temperature, and pulse rate. Nickerson et al [21] predicted pain scores—measured between 40 and 120 minutes after administering 10 mg of oxycodone—from pain score values before drug administration using 200 features for each patient in the electronic medical records data. Essential features included age, gender, Charlson comorbidity index, BMI, ethnicity, and International Classification of Diseases ninth edition code class. They predicted a postmedication (oxycodone) pain score with an accuracy of 66% using support vector regression. They concluded that these results would likely improve with more temporal data (eg, vital signs), which we explore in this study by using both vital signs and medication data to predict the severity of pain with multiple Likert scales, with the best performance of 82.8% accuracy using only 9 features, as shown in Textbox 1. Although most prior studies have explored this question in the context of misuse of pain medications (particularly regarding abuse of opioid medications), we used the pain medication provided to patients to predict the severity of pain. The ability to objectively and accurately predict pain severity and onset could result in more prompt and effective treatment of pain crises, leading to improved outcomes and encouraging more diligent use of medications [22]. Although there are complications associated with using medication data for prediction at the same time as pain measurements, we investigated their application here to provide a baseline for this comparison and open the door for future research involving dynamic pain and medication measurements. Our principal hypothesis was that pain medications help with better pain-related function and pain intensity management.

Several previous studies from our research group used EHR data to predict pain. Yang et al [4] initially demonstrated the feasibility of ML techniques on a limited data set of 5363 records from 40 patients during inpatient hospital visits to predict subjective pain scores from 6 objective vital signs with support vector machines (SVMs), achieving an accuracy of 58.2%. Alambo et al [8] examined 424 clinical notes from the same cohort of 40 patients to predict the prevalence of pain and whether pain increased, decreased, or remained constant. Padhee et al [6], using 6 objective vital signs and the nature of hospital visit information from 59,728 records of a different cohort of 47 patients over 5 consecutive years, demonstrated that with more data for each patient, the accuracy for pain prediction improved (accuracy of 65.3%). In this study, we demonstrated that with more data from a larger cohort of patients and medication information, the accuracy improved by 17.5% compared with Padhee et al [6] and by 24.6% compared with Yang et al [4].

Deep neural networks have been shown to contribute promising capabilities in learning complex patterns in data and have achieved remarkable success in several domains such as computer vision, natural language processing, and speech recognition. Recently, many efforts have been made to improve the performance of ML tasks in the field of biomedical and health informatics. Deep learning has previously been successfully used on EHRs to achieve both specific and general goals [23]; for instance, both *Deep Patient* [24] and *Doctor AI* [25] used unsupervised deep learning before supervised learning. As in many other applications, the challenge of missing data is common in ML studies applied to EHR data, which often contain entries with missing elements. These challenges arise as the data are manually collected from patients and may vary depending on circumstances. In this study, we address this challenge using variational autoencoders (VAEs) [26,27] to reconstruct missing data. VAEs are unsupervised deep feature methods that provide data reconstruction by probabilistically filling in the data between the encoding and decoding steps. As the encoder neural network typically expects a fixed-length vector as input, the question arises regarding what we can do with the missing values in the VAE encoder input. We followed the heuristic of replacing missing elements with fixed values [28,29]. Although VAEs have been previously applied to EHR data [30], we show here that they improve pain prediction capabilities from physiological signs with and without medication information.

**Data modalities and variables considered in this study.**

**Vital signs**

- Peripheral capillary oxygen saturation
- Systolic blood pressure
- Diastolic blood pressure
- Heart rate
- Respiratory rate
- Temperature

**Medication**

- Medication type (5 classes)
  - Hydromorphone
  - Acetaminophen
  - Ketorolac
  - Oxycodone
  - Fentanyl
- Medication status (2 classes)
  - Given or applied
  - Missed or removed or due
- Total medication dosage (mg/mL)

**Pain**

- Self-reported pain score on a scale of 0-10 (0=no pain to 10=severe and unbearable pain)

### Objective

This study aimed to use vital signs and medication information collected from the EHR data of patients with SCD to predict patient-reported pain scores using ML techniques. In this paper, we propose to represent multiple data modalities in EHRs in high-level abstraction, vital signs, and medication information using deep autoencoder networks such as VAEs to predict pain intensity on varying Likert scales. Our specific contributions are as follows:

1. To the best of our knowledge, we analyzed the most extensive EHR data of 126,519 records from 496 patients with SCD collected over 5 consecutive years and demonstrated that a larger patient cohort data improves model performance in pain prediction. We reduced the data to 33,000 records by removing data with multiple medication fields missing and then used them for our model evaluation.
2. We showed that pain medication information with vital signs data can improve pain prediction at varying pain rating scales (ie, different granularities).
3. We demonstrated that deep representational learning can not only improve pain prediction results but also provide a better understanding of the role of medication and physiology on the patient’s pain response with a patient profiles study.

## Methods

### Data

In this study, we analyzed inpatient and outpatient EHR data collected from 496 participants at Duke University Hospital over 5 consecutive years. Each record contained measures of 6 vital signs, as shown in Textbox 1. In addition to the vital signs, each record also included the patient’s self-reported pain score, with an ordinal range from 0 (no pain) to 10 (severe and unbearable pain). The pain score was recorded by the medical staff during outpatient visits when the patients reported no pain (pain score 0) and during their monitoring of inpatient visits. The vital signs were recorded by the medical staff every 4 hours for inpatient stays, and the medication data were recorded as given to the patients. We extracted 3 medicinal features from the data upon consultation with our coauthor physician, as shown in Textbox 1. We calculated the total medication dosage as the sum of all medication dosages recorded for a patient at a given time *t* using the following equation:



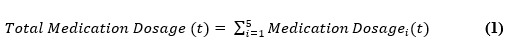



Here, *Medication Dosage*_i_(*t*) indicates the dosage of *i*th medication type recorded at time *t* for the patient.

We removed the data points in this study for which the Medication Administration Record was on hold.

### Background

In this study, we used VAEs to impute missing values within the data based on other samples. Autoencoders are a class of unsupervised deep learning techniques in which neural networks are leveraged for the task of representation learning. We designed a neural network architecture to impose a bottleneck in the network, thereby forcing a compressed knowledge representation of the original input data modalities. If the input features were such that they were independent of one another, this compression and subsequent reconstruction would be an arduous task. However, if some association exists in the data (eg, correlations between input data modalities), this structure can be learned and consequently leveraged when forcing the input through the network’s bottleneck. VAEs are probabilistic generative models that have the same architecture as vanilla autoencoders but consider specific assumptions about the distribution of middle or latent layer variables. They learn the true distribution of input features from latent variable distributions using a Bayesian approach and present a theoretical framework for reconstruction and regularization [31].

**Figure 1:**
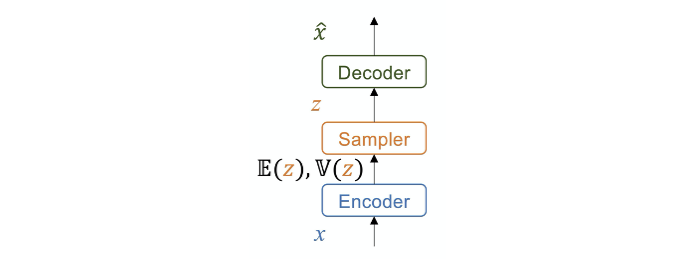
An illustration of the variational autoencoder architecture for one input data modality.



A VAE learns the distribution of data with an encoder network by fitting it to a Gaussian distribution and generates data with a decoder by sampling from the learned distribution. We used autoencoders to reconstruct the input data (*x*) in the output 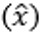 layer by an encoding and decoding process. As shown in Figure 1, the encoder network converts the input data (*x*) into a latent representation (*z*). The hidden state comprises 2 additional layers: *E*(*z*) and *V*(*z*), where the latent variable *z* follows a Gaussian distribution with mean *E*(*z*) and variance *V*(*z*). We sample *z* from the distribution parameterized by the encoder; the decoder network then remodels the input from the latent representations by using *z* to generate 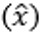. The fundamental property of autoencoders is that they minimize this reconstruction error using a loss function comprising a reconstruction term (*l_reconstruction_*), which is the mean squared error between the output and the input, and a regularization term (Kullback-Leibler divergence loss [*l_KL_*]), as shown in the following equation:

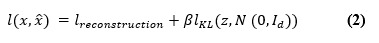



The term 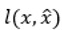 is on the final layer and the regularization term enforces a specific Gaussian structure on the latent layer through a penalty term *l*_KL_(*z*, *N* (0, *I*_d_)). β in the loss function is a hyperparameter that dictates how to weigh the reconstruction and penalty terms.

Variation in VAE means that the encoder network estimates the μ (mean) and σ (SD) parameters (latent variables) of the Gaussian distribution. However, real-world applications, including health care, almost always have missing values. In correspondence with the missing values in the raw temporal data, we substituted the corresponding categories with a unique integer to properly encode the status of the missing information. The encoder comprises a long short-term memory cell. It receives input sequences resulting from the concatenation of the raw physiological data and the extracted categorical medicinal features. As in every encoder in a VAE architecture, it produces an output that is used to approximate the mean and variance of the latent distribution. The decoder samples from the latent distribution form the output sequences. This approach helps us develop an unsupervised framework that can fill the missing pieces appearing in real-world EHR data volume streams, not only in patients with SCD but also in other health care applications.

### Proposed Framework

#### Overview

**Figure 2:**
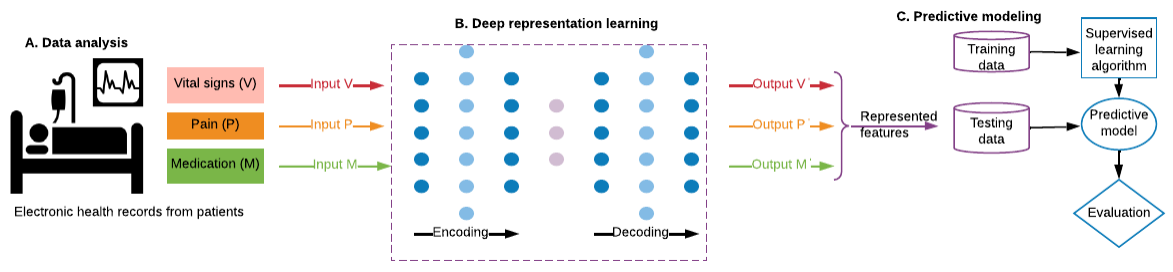
Deep representation learning for pain prediction (A: data analysis, B: deep representation learning, and C: predictive modeling).

Figure 2 provides an overview of the proposed approach in 3 consecutive steps. In step A, we preprocessed the raw data to overcome data challenges such as missing values. Next, in step B, we applied unsupervised deep representation learning to generate higher-level abstraction of the input data modalities. Finally, in step C, we investigated supervised algorithms for predictive modeling and performed the evaluation.

#### Step A: Data Analysis

In this study, we used records from EHR data collected at Duke University Hospital and identified them using study labels to label patients without identification. The timestamp for each data entry was deidentified, preserving temporality. The data set had missing values for ≥1 vital sign, medication, and pain score. The data set contained 126,519 records from 496 patients collected over 5 consecutive years. However, we included 33,000 records in this study owing to >4 missing features in the remaining records. Of the 33,000 records, 18,291 (55.43%) included at least 1 of the 5 administered medication types (Textbox 1). The demographic information of the patients was not available. Data for each patient varied; although 70 patients had a one-time visit to the hospital, 240 patients visited for at least >100 days. Most patient records were for a patient staying for 1705 days with a high mean pain score of 8 who received pain medication 219 times (an average of 338 mg of total pain medication dosage). We did not consider the effect of any other medical condition on the patients in this study.

#### Step B: Deep Representation Learning

In the second step, we represented all input data modalities in high-level abstraction using multiple deep autoencoder networks, including VAEs. We evaluated the performance of each network while considering the tuning of hyperparameters such as the learning rate, batch size, number of epochs, and number of hidden layers and hidden units for training to avoid overfitting.

#### Step C: Predictive Modeling

In this step, we applied supervised learning techniques to the represented data set using linear and nonlinear approaches such as random forest (RF) [32], Lasso regression [33], and SVM [34]. Our experiments comprised three main phases: (1) training the VAE, where the training samples were used to train the VAE and the reconstruction loss for each training data sample was stored according to the target pain score; (2) generating new pain scores, where the VAE decoder generated new pain score samples based on specified classes and each newly generated data sample was merged into the original training data set under the condition that the class reconstruction loss was satisfied; and (3) predicting pain scores, where the VAE decoder was used to initialize the weight of the hidden layers, the merged training data set was used to train the classifier, and the trained classifier was used to predict pain scores on the testing data set.

### Experimental Study

In our experimental study, we implemented our methodology on a deidentified EHR data set. This study design helped us discover our method’s performance in predictive modeling for patients with SCD. Across several attributes comprising patient clinical records and individual health status, 9 attributes, including vital signs and medication information, were considered for the data analysis of 496 patients. As mentioned previously, the goal was to predict pain scores based on high-dimensional features.

We implemented the VAE using the *PyTorch* and *Keras* libraries with a *TensorFlow* backend in Python. The VAE architecture has 5 hidden layers (2 hidden layers of encoders and decoders and 1 middle layer). We applied hyperparameter tuning for major hyperparameters such as the learning rate, activation functions, and batch size to select the best hyperparameters. We used a hidden dropout component with a dropout rate of 0.2 and a sigmoid activation function for the final layers. The models were trained for 100 epochs using an Adam optimizer with a learning rate of 0.001 (with exponential decay rates of first- and second-moment estimates β_1_=.9 and β_2_=.999) and a batch size of 64. Once the latent features were extracted, they were fed into a supervised learning model for pain score prediction. For the supervised learning step, we considered 3 well-known supervised classifiers: RF (with 50 trees and half of the features considered at every split), Lasso regression, and SVM (with radial basis function kernel C 1.5 and gamma set to 1/N_f_, where N_f_ denotes the number of features). We used the grid search method to determine the optimal hyperparameters for supervised classifiers. We used the average accuracy as our evaluation measure for performance evaluation in the testing process. Finally, we visually inspected the learned representations of the entire data set and compared them with the represented data. We used t-distributed stochastic neighboring embedding (t-SNE) [35] for this task.

### Ethics Approval

The study protocol was approved by the institutional review board of Duke University Medical Center in May 2018 with IRB number Pro00068979. Identifiable personal information was not collected; all data were kept confidential and safe according to the internal data security policy, and they were only accessible to authorized researchers.

## Results

### Vital Signs

We evaluated our approach using VAE data (represented data) and original data (unrepresented data) on supervised classifiers for pain prediction tasks and compared their performance based on the results obtained from the testing process with 5-folds cross-validation (for each fold, we considered 80% of the data for training, 10% for the validation set, and 10% for the test set). This comparison is presented in Table 1. We would like to note here that missing values for pain scores were not imputed. In our data set, we had 11 unique self-reported pain scores where patients described their experienced pain intensity on a scale of 0 to 10. It is challenging for one person to distinguish between such broad and granular pain intensity levels and be consistent in the reporting of every pain episode. Hence, in addition to the 11 pain scores, we evaluated our pain prediction models by transforming our data set into a 6-point rating scale, a 4-point rating scale, and a binary rating scale according to the following transformation rules:

1. The 6 pain scores: none=0, very mild=1 to 2, mild=3 to 4, moderate=5 to 6, severe=7 to 8, and very severe=9 to 10
2. The 4 pain scores: none=0, mild=1 to 3, moderate=4 to 6, and severe=7 to 10
3. The 2 pain scores: no or mild pain=0 to 5 and severe pain=6 to 10

As shown in Table 1, an RF-supervised classifier trained on data represented using VAE performed best in each pain rating scale, achieving the highest accuracy of 60.3% in predicting 2 pain scores (no or mild pain and severe pain). According to these results, our approach with representation learning reduces the prediction error and achieves better accuracy than using the original features.

**Table 1:** Pain prediction results in varying pain scales on vital signs data (accuracy) arranged from higher resolution to lower resolution.

Approach	11 pain scores			6 pain scores			4 pain scores				2 pain scores		
	RF^a^	SVM^b^	Lasso	RF	SVM	Lasso	RF	SVM	Lasso	RF		SVM	Lasso
Original data	0.301	0.242	0.216	0.363	0.352	0.337	0.432	0.426	0.392	0.535		0.513	0.486
VAE^c^ data	0.343	0.321	0.307	0.391	0.371	0.348	0.472	0.452	0.439	0.603		0.561	0.549

^a^RF: random forest.

^b^SVM: support vector machine.

^c^VAE: variational autoencoder.

### Vital Signs and Medicinal Data

We also analyzed the performance of pain score prediction using only vital signs compared with including medication information. We show in Table 2 that our approach with the RF classifier achieves better accuracy with medication and vital signs information than with only vital signs information in predicting the respective pain scores. This indicates that when provided with additional medication information, our approach can learn better representations of patient profiles from vital signs to predict their pain levels. The higher accuracy associated with the narrow scales is attributed to the narrow space to misclassify many records by our models, thereby improving the chances of correctly predicting the pain score.

We also show in Table 3 the area under the curve (AUC) for the receiver operating characteristic for the best-performing, clinically relevant (as suggested by our coauthor clinical partner) models (models a, b, d, and e from Table 2). Overall, the AUC for both the 2 pain score and 4 pain score rating scales suggested no discrimination. This indicates that our models can predict pain in patients based on their vital signs and medication information at various intensity levels. For the 2 pain score rating scales, an AUC of 0.92 suggests a 92% chance that our model correctly distinguishes a pain score in the no or mild pain range (0-5 pain score) from the severe pain range (6-10 pain score) based on the patient’s vital signs and medication information instead of a random assignment probability of 50%. Empirically, our results demonstrate that (1) medical feature representation can improve prediction performance, and (2) medication information can lead to significant improvement in pain level prediction.

**Table 2:** Pain prediction results in varying pain scales on vital signs data (accuracy) as compared with additional medication data arranged from higher resolution to lower resolution.

Approach	11 pain scores			6 pain scores			4 pain scores			2 pain scores	
	Vitals	Vitals+medicinal	Vitals		Vitals+medicinal	Vitals		Vitals+medicinal	Vitals		Vitals+medicinal
Original data	0.301	0.442	0.363		0.463	0.432		0.689^a^	0.535		0.787^b^
VAE^c^ data	0.343	0.476	0.391		0.493	0.472		0.706^d^	0.603		0.828^e^

^a^Model with original representations using both vital and medication data for the 4 pain score rating scale.

^b^Model with original representations using both vital and medication data for the 2 pain score rating scale.

^c^VAE: variational autoencoder.

^d^Model with deep representations using both vital and medication data for the 4 pain score rating scale.

^e^Best-performing model with deep representations using both vital and medication data for the 2 pain score rating scale.

**Table 3:** Area under the curve for the receiver operating characteristic for the best-performing models (models a, b, d, and e).

Approach	4 pain scores					2 pain scores	
	None: 0	Mild: 1-3	Moderate: 4-6	Severe: 7-10	No or mild pain: 0-5		Severe pain: 6-10
Original data	0.82	0.85	0.88	0.83	0.91		0.89
VAE^a^ data	0.82	0.86	0.89	0.83	0.92		0.89

^a^VAE: variational autoencoder.

## Discussion

### Principal Findings

#### Overview

Our study demonstrates that although there are complications associated with using medication data for prediction at the same time as pain measurements, ML models can be used for dynamic pain and medication measurements. Our findings indicate the importance of medication information (achieving an accuracy of 82.3%) and demonstrate that a larger cohort of patient data with deep representational learning improves model performance (by 17.5% as compared with Padhee et al [6] and by 24.6% as compared with Yang et al [4]). Furthermore, from our unsupervised analysis, we distinguished unique patient profiles (Table 4) that can help isolate different patient profiles to further understand the role of physiology and medication in pain response. In addition, there are 2 main types of opioids: short-acting analgesics and sustained-release analgesics, and the dosing pattern differs depending on the properties of these drugs. Our initial results show that considering medication type (Textbox 1), status, and dosage can improve pain assessment models, providing evidence for future studies to further analyze the variability in dosing patterns.

**Table 4:** Sample of patient profiles from the learned variational autoencoder representations clustered together using t-distributed stochastic neighboring embedding projections, as shown in Figure 3.

Medication administered	Region	Patient number	Correlation between medication and pain score	Vital signs	Correlation between medication dosage and vital signs
Oxycodone	1^a^	1	0.23	Temperature	0.65
Hydromorphone	1	1	0.11	Temperature	0.65
Acetaminophen	1	1	0.17	Temperature	0.65
Ketorolac	1	2	0.55	Systolic blood pressure	0.47
Hydromorphone	1	2	0.24	Systolic blood pressure	0.47
Hydromorphone	2^b^	3	0.35	Systolic blood pressure	0.099
Acetaminophen	2	3	–0.20	Systolic blood pressure	0.099
Ketorolac	2	3	0.41	Pulse	0.04
Oxycodone	3^b^	4	0.59	Systolic blood pressure	0.27
Hydromorphone	3	4	0.08	Systolic blood pressure	0.27
Fentanyl	4^c^	5	—^d^	Peripheral capillary oxygen saturation level	0.13
Acetaminophen	4	6	—	Temperature	0.47
Acetaminophen	4	6	—	Pulse	0.38
Acetaminophen	4	6	—	Peripheral capillary oxygen saturation level	0.28

^a^High pain.

^b^Moderate pain.

^c^No or low pain.

^d^Medication not available.

**Figure 3:**
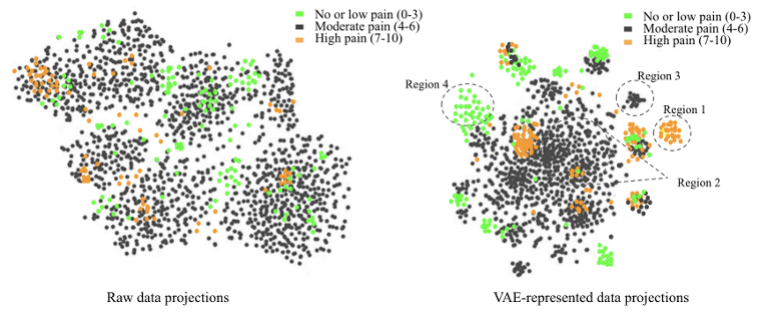
Visualization of the learned data representations using t-SNE: t-distributed stochastic neighboring embedding projections. VAE: variational autoencoder.

#### Deep Representation Learning

**Figure 4:**
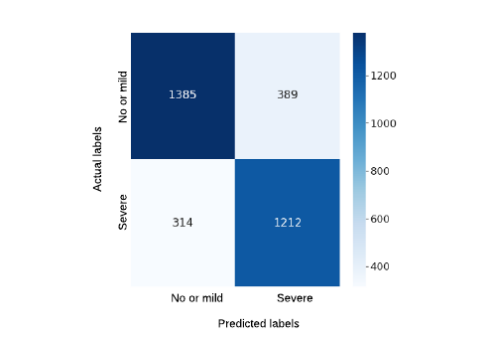
Confusion matrix for the best-performing model with original data representations for 2 pain score levels (pain scores: no or mild=0-5 and severe=6-10; Table 2, model b).

**Figure 5:**
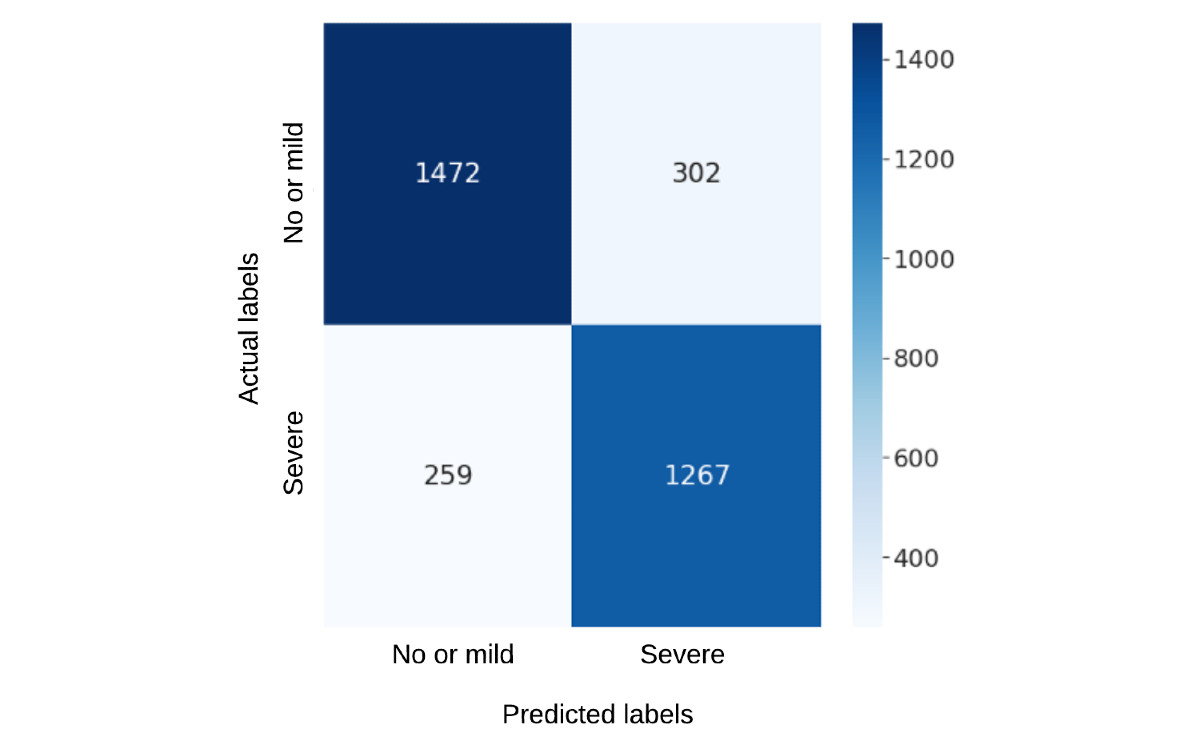
Confusion matrix for the best-performing model with variational autoencoder data representations for 2 pain score levels (pain scores: no or mild=0-5 and severe=6-10; Table 2, model e).

**Figure 6:**
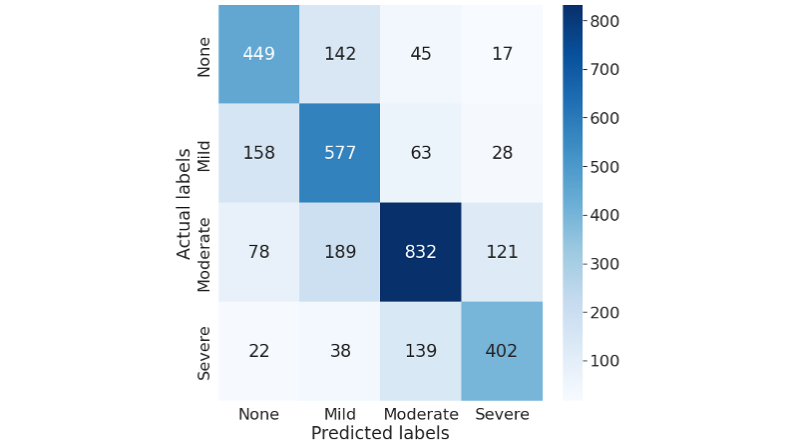
Confusion matrix for the best-performing model with original data representations for 4 pain score levels (pain scores: none=0, mild=1-3, moderate=4-6, and severe=7-10; Table 2, model a).

**Figure 7:**
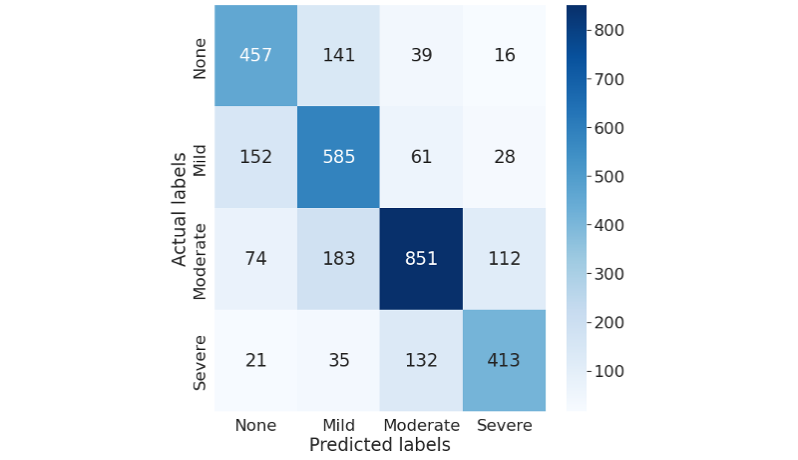
Confusion matrix for the best-performing model with variational autoencoder data representations for 4 pain score levels (pain scores: none=0, mild=1-3, moderate=4-6, and severe=7-10; Table 2, model d).

In this study, we applied a deep feature representation to predict the pain scores of patients with SCD based on their vital signs and medication information. These results emphasize that representation learning can play an effective role in the performance of clinical prediction. As shown in Table 1, our models trained on deep represented features can identify pain scores for 6.8% more patients at an abstract pain intensity level of no or mild pain or severe pain. They also display significant improvement by detecting pain intensity for 4.2% more patients at a highly granular pain score intensity (ie, on 11 pain ratings) than models trained on unrepresented raw vital signs data. We observed a similar performance of deep represented features compared with raw data features when medicinal data were included in the modeling, which may indicate that the medication information can allow the use of simpler features by providing more pain-related information than the more convoluted deep represented features. However, our models trained on VAE-represented features generated using both vitals and medicinal data could identify pain scores for 3.4%, 3%, 1.7%, and 4.1% more patients from higher to lower resolutions of pain intensity than models trained on raw data. To investigate further, we show the confusion matrices of the best-performing models trained on vitals and medicinal data in Figures 4-7. As shown in Figures 4 and 5, it is interesting to note that with deep feature representations, our model can accurately identify not just 87 more cases of no or mild pain but 55 more cases of severe pain while reducing misclassification. This is important to consider in a clinical setting while deciding on the diligent use of medications in a larger patient cohort.

Similarly, Figures 6 and 7 show that with more granular 4-point pain intensity levels (pain scores: none=0, mild=1-3, moderate=4-6, and severe=7-10), our model trained on deep represented features can identify more instances for each category accurately than the original data representations while reducing the misclassification. The model can identify more instances of moderate pain than none, mild, or severe pain. It is noteworthy that the misclassification for each pain category reduced with the stretch between the pain severity levels. For example, as shown in Figure 7, our best model for 4 pain scores (Table 2) incorrectly predicted 21 instances of severe pain data as no pain, 35 instances as mild pain, and 132 instances as moderate pain, highlighting that the error primarily lies in the prediction of moderate pain as no pain. Similarly, it predicted 16 instances of low pain as severe pain, 39 as moderate pain, and 141 as mild pain. Misclassification reduces with the granularity of pain intensity, reflecting the subjective nature of pain.

#### Role of Medicinal Data

Although prior studies have shown the efficacy of data mining techniques in implementing medical decision-making with treatment outcome prediction [36,37], to the best of our knowledge, this is the first study to analyze the role of medication in pain level prediction for patients with SCD. Our results show that for abstract pain levels, representational learning–based approaches can predict whether a patient is experiencing pain for 22.5% more patients when provided with their medication information (Table 2). This means that when our model is provided with not only vital signs information but also medication type, total medication dosage, and whether the medication was given or missed, it can better predict whether more patients are experiencing pain than when provided with only vital signs. This finding is substantiated by the current medical literature on pain management [38], where clinical research focuses on determining the optimal medication dosage for individual patients. By building a model that incorporates medication information and physiological data, we are one step closer to future pain forecasting that can use current physiological information and pain medication to predict pain at a future time point for assessing the next medication dosage and time.

Furthermore, for a higher resolution of pain levels (ie, 11 levels), our deep representational learning–based approaches could predict subjective pain scores for 13.3% more patients when provided with medication information. In addition, our model can predict such highly subjective pain scores for 38.5% more patients than the random assignment of 9.09% (ie, 1/11 pain scores) when provided with both vital signs and medication information of the patients.

### Visualization

We visually inspected the learned representations of the entire data set obtained from VAE representations. Using t-SNE plots, as shown in Figure 3, we compared the disentanglement levels of the represented and raw data. The t-SNE projections clearly show that the VAE can produce sparser and more disentangled representations than the raw data. Although the t-SNE projections of the raw data also indicate data separability, the deep representations can identify variations in mean pain scores (low, moderate, and high). This may explain the competitive performance of the benchmark classifiers in the previous section and the advantage of integrating vital signs and medication data. Although some embeddings were clearly clustered closer to the same pain range, we also observed some overlaps. Specifically, we observed better alignment among the low pain and high pain profiles than among the moderate pain profiles. This may be because of the variation and frequency of the data recordings made for the patients. These preliminary visualization results indicate that our VAE method may require additional data to generate representations that obtain a more granular separation between patients’ pain scores.

### Patient Profiles

To understand the alignment of the representations learned by our best-performing VAE model, we illustrated 6 sample patient profiles clustered into the 3 pain range categories (no or low=0-3, moderate=4-6, and high=7-10) by the t-SNE projections of the embeddings (as shown in Figure 3). As shown in Table 4, we present 2 patient profiles from each of the 3 categories of pain scores with regard to the medication administered and the vital signs. It should be noted that we specifically chose regions where the pain profiles belonged to 1 of the 3 pain levels. Although we chose 2 patient profiles from better-aligned regions 1 and 4, we compared 2 patient profiles from a more spread-out moderate pain intensity (regions 2 and 3). Patient numbers are anonymized patient identifiers used in this study.

**Figure 8:**
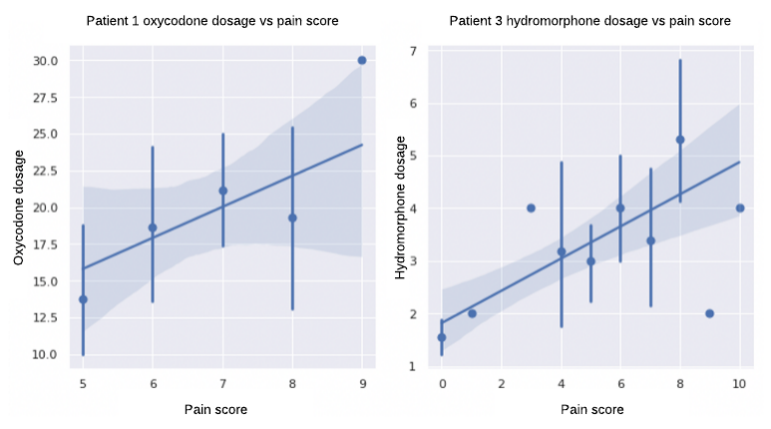
Distribution of medication dosage with pain score for sample patients with high and moderate mean pain intensity.

We observed a positive correlation between the medications administered and pain scores in all 4 patients with high and moderate pain levels. This reflects that the patients reporting higher pain scores were administered an increased medication dosage (as shown in Figure 8 for patients 1 and 3), and our model learned that relationship. For both patients (patients 1 and 2) with high pain, we observed a positive correlation between hydromorphone dosage and pain score, as well as a high correlation between total medication dosage and vital signs, which may be reflective of more pain medications being given when a patient has high pain. This indicates that our model learned the interplay among medications, vital signs, and pain intensity. It will be interesting to analyze these correlations before and after medication in the future.

For both patients (patients 3 and 4) with moderate pain, in addition to a positive correlation between hydromorphone and pain score, we observed a positive correlation between medication and blood pressure. This indicates that our model learned a possible association of administering hydromorphone for moderate pain intensity levels, during which the patients have elevated blood pressure. However, patient 4 had a higher positive correlation between medication dosage and vital signs than patient 3. This may be a possible reason that they were not close in the embedding space and belonged to distant regions, as shown in Figure 3.

Although we did not observe any significant correlation between medication and pain scores for both patients (patients 5 and 6) with no or low pain, we observed a positive correlation between medication and vital signs. This may again be suggestive of elevated vital signs that occur with pain, leading to medication administration. Although both patients might have reported varying pain scores between 0 and 3, it is highly challenging to differentiate between pain scores of 1 and 2 or 2 and 3. Hence, it might be the case that with medication, their vitals improved (as indicated by the positive correlation), making them feel better. This sample patient profile study indicated that deep feature representations can be used to learn complex relationships between various factors influencing pain management. With more data for each patient, this study can be extended to the design of personalized pain management tools to assist clinicians.

### Study Strengths

The design of an objective pain prediction model could potentially assist medical providers in pain management. The lack of objective pain markers has limited the optimal pain assessment strategies for patients with regular pain episodes. As discussed earlier, objective vital signs data can improve pain assessment using ML algorithms. In this study, we developed ML models that can classify pain scores of patients at varying scales and may soon be used to predict pain intensity in individuals with pain based on objective and physiological data and the type, dosage, and status of medication. In the future, a tool designed using our model could be used reasonably quickly to generate pain intensity predictions for unseen new patient data in both inpatient and outpatient hospital visits. This research provides an essential step toward assisting medical practitioners with additional objective pain measures while deciding on a pain management strategy.

### Limitations

There were a few limitations to our study. We did not consider our hypothesis that each patient had pain management strategies through individualized pain protocols, which varied among patients and led to specific pain medications being administered at varying intervals. In addition, because of variations in patient pain record intervals, we did not evaluate before and after administration of pain medication in pain prediction. Both opioid and nonopioid medications, when administered, are known to affect vital sign parameters independently and to varying degrees. For example, opioids can slow a patient’s breathing and lower blood pressure. Furthermore, the status of medicine prescription and total medication dosage are subjective variables that may vary between centers and physicians. Our analysis using these variables has not been validated using data from multiple centers.

Furthermore, pain medications may affect patients to different degrees based on the dosing, type of medication, and previous patient history of receiving pain medications. Owing to the variation in data per patient, we could not evaluate such individualized factors. In the future, it will be helpful to analyze the role of individual medication protocols in individualized pain prediction and pre- and postadministration changes.

### Conclusions

In this study, we propose an effective pain prediction model based on objective vital signs and pain medication use. Our experiments demonstrated that information about pain medication (type, dosage, and status) can improve pain intensity prediction at both abstract and granular levels. Our analysis indicates the role of medication information in pain assessment and demonstrates that a larger cohort of patient data with deep representational learning improves model performance and can help isolate different patient profiles for further understanding of the role of physiology and medication on pain response. In the future, this study can be extended to further investigate the effect of variation in medication protocols, such as changes in vital signs before and after medication and the time elapsed between medication doses. This would be an essential part of a real-time pain forecasting system and can be extended as a trial that evaluates the timing of the administration of additional doses of opioids based on physiological and objective data alone. Our initial results indicate promise in pursuing each of these efforts, and our study is a valuable addition to ongoing studies investigating how objective vital signs and medication data can be used to help providers to better understand and design pain management strategies.

## Abbreviations

AUC: area under the curve
EHR: electronic health record
ML: machine learning
RBC: red blood cell
RF: random forest
SCD: sickle cell disease
SD: Standard Deviation
SVM: support vector machine
t-SNE: t-distributed stochastic neighboring embedding
VAE: variational autoencoder
